# Effect of unripe banana flour as a functional feed ingredient on growth performance, internal organ relative weight and carcass traits of broilers

**DOI:** 10.1002/vms3.1070

**Published:** 2023-01-11

**Authors:** Oktafia Munita Rahmawati, Sugiharto Sugiharto, Turrini Yudiarti, Endang Widiastuti, Hanny Indrat Wahyuni, Tri Agus Sartono, Ikania Agusetyaningsih, Tugay Ayasan

**Affiliations:** ^1^ Department of Animal Science, Faculty of Animal and Agricultural Sciences Universitas Diponegoro Semarang Central Java Indonesia; ^2^ Kadirli Faculty of Applied Sciences, Department of Organic Farming Business Management Osmaniye Korkut Ata University Osmaniye Turkey

**Keywords:** banana flour, broilers, feed conversion, functional feed, meat quality

## Abstract

**Background:**

Following the prohibition of in‐feed antibiotics, poultry nutritionists are increasingly interested in the use of functional feed. Unripe banana flour (UBF) contains significant amounts of oligosaccharides (which may act as prebiotics) and antioxidants, making it a potential functional feed for broilers. However, research on the use of UBF as a functional feed ingredient for broilers is limited.

**Objectives:**

The study investigated the effect of UBF with or without probiotic and multienzyme on growth, internal organ weight and carcass characteristics of broilers.

**Methods:**

A total of 392 broiler chicks were distributed into 4 groups included CONT (chicks receiving control feed), UBF (chicks receiving 5% UBF in feed), UBFPRO (5% UBF plus 0.05% probiotics) and UBFZYM (5% UBF plus 0.05% multienzyme). Data on growth performance were weekly recorded, whereas data on internal organs and carcass were collected on day 38.

**Results:**

Feed conversion ratio (FCR) was lower (*p* < 0.05) in UBF, UBFPRO and UBFZYM than that in CONT chicks, with no significant difference in body weight, body weight gain and feed intake. There was a tendency that gizzard was higher (*p* = 0.08) in CONT than in UBF chicks. Also, pancreas tended (*p* = 0.09) to be lower in UBFZYM than in CONT birds. There was a notable effect (*p* < 0.05) of dietary treatments on the yellowness (b*) values of thigh meats, in which UBFPRO had lower b* values than that of CONT but did not differ from that of UBF and UBFZYM. There was no difference (*p* < 0.05) in carcass and commercial proportion of broilers.

**Conclusions:**

Feeding of 5% UBF with or without probiotic and multienzyme improved FCR, without negatively affecting the carcass characteristics of broilers.

## Graphical abstract

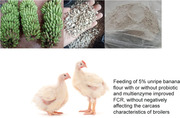

## 1 INTRODUCTION

Broiler chicken is one of the most important livestock commodities for supplying animal protein to people. Broiler industry is also crucial to the economy of many countries. Hence, the sustainability of the broiler farming is very critical for the fulfilment of nutrition as well as the economy of the country. Antibiotic growth promoters (AGP) have widely been utilized in the broiler industry for decades to maximize growth potential and health. However, most countries, including Indonesia, have outlawed the use of AGP for food safety reasons. Indeed, excessive and continuous use of AGP in broiler chickens can result in AGP residues in the meat, posing a health risk to consumers (Sugiharto, 2016). The use of functional feed is increasingly attracting the attention of poultry nutritionists following the prohibition of AGP on broiler production. Besides containing nutrients as conventional feedstuffs, functional feed contains various bioactive compounds that can have promoting impacts on the health and growth of broilers (Sugiharto et al., 2018). Moreover, functional feed could improve the carcass traits (Alwaleed et al., 2020) and meat quality of broilers (Nopparatmaitree et al., 2022).

Study has shown that unripe (green) banana flour, in addition to having a high energy (Dumorné et al., 2020), contains substantial amount of oligosaccharides and resistant starch (Chang et al., 2022). These active components are often attributed to prebiotics, which can improve intestinal ecology and function of poultry. Unripe banana flour (UBF) also showed a high antioxidant activity (Chang et al., 2022; Padam et al., 2014), which is essential in improving the physiological condition and health of broilers reared under intensive system. In line with the above studies, our recent study showed that four cultivars of UBF in Indonesia contained energy exceeding 3000 kcal/kg (Munita et al., 2022). These data confirm that UBF can be used as an alternative energy source to replace yellow corn, the price of which is very volatile. Our data also showed that UBF has prebiotic activity that can support the growth of probiotic bacteria (*Lactobacillus casei*). Also, UBF showed a high antioxidant activity, as indicated by the ability of UBF in scavenging the 2,2‐diphenyl‐1‐picrylhydrazyl (DPPH) (Munita et al., 2022). The use of UBF as a feed ingredient for broilers is currently still lacking. To the best of our knowledge, only Dumorné et al. (2020) used UBF as the energy source for broiler chickens. They confirmed that the inclusion of banana flour into broiler feeds up to 20% had no adverse effect on broiler growth performance.

UBF is generally produced from the discarded/culled green bananas during fruit selection and processing. In some banana‐producing countries, culled green bananas are so abundant that, if not utilized, they can cause environmental problems (Padam et al., 2014). In addition to banana cultivars commonly consumed by humans, several types of bananas are not favoured by consumers because they have a sour and unpleasant taste. As a consequence, these cultivars of banana have low economic value (Munita et al., 2022). Taking these above facts into consideration, the application of UBF as functional feed ingredients may therefore increase the economic worth of the underconsumed banana cultivars as well as support the sustainability of broiler production. Probiotics and enzymes are two types of feed additives that have widely been applied to broiler chicken production. Besides being able to work individually, probiotics are reported to be able to synergize with prebiotics to further improve intestinal function and growth performance of broiler chickens (Sugiharto, 2016). The synergistic effect was also seen between prebiotics and enzymes in improving broiler production performance as confirmed by Shang and Kim (2017). In this study, UBF was included into broiler feeds in conjunction with probiotic *L. casei* and multienzyme. The aim of the present study was to investigate the effect of UBF as a functional feed ingredient with or without probiotic and multienzyme on growth performance, internal organ relative weight and carcass characteristics of broilers.

## 2 MATERIALS AND METHODS

### 2.1 Preparation of unripe banana flour

The underconsumed banana cultivar (“Norowito” cultivar), which has a sour and unpleasant taste (Munita et al., 2022), was used to produce UBF in the present study. Unripe banana (green banana) was collected from the local banana plantation. The unripe banana was peeled, washed, chopped into small pieces and sun‐dried. The unripe banana pieces were then mashed and sifted (2 mm) to produce UBF. Sample of UBF was obtained for proximate analysis according to the standard AOAC method (AOAC, 1995). The gross energy content in UBF was determined using a bomb calorimeter (Parr Instrument Co., Moline, IL, USA), of which benzoic acid was used as a calibration standard. The rest of the sample was also analysed for antioxidant activity based on the DPPH method as described by Wu et al. (2009) with few modifications. The pH of UBF was determined using pH meter (Portable pH Meter OHAUS ST300). The analysis/measurement was conducted in duplicate. The data on chemical composition, antioxidant activity and pH value of UBF are listed in Table 1.

**TABLE 1:** Chemical composition, antioxidant activity and pH value of unripe banana flour

**Items**	**Values (means ± standard deviations)**
Moisture (%)	10.4 ± 0.30
Crude protein (% DM)	3.89 ± 0.01
Crude fibre (% DM)	3.51 ± 0.30
Ash (% DM)	3.82 ± 0.06
Crude fat (% DM)	0.32 ± 0.06
Carbohydrates (% DM)	81.4 ± 0.03
Total energy (kcal/100 g)	344 ± 0.51
Energy from fat (kcal/100 g)	2.88 ± 0.57
DPPH scavenging activity (%)	94.4 ± 0.21
pH	6.04 ± 0.02

Abbreviations: DM, dry matter; DPPH, 2,2‐diphenyl‐1‐picrylhydrazyl.

### 2.2 Broiler chicken experiment

The in vivo experiment was designed according to a completely randomized design. A total of 392 Cobb (unisex) broiler chickens were reared communally using commercial prestarter feed (according to feed label, containing 22%–24% crude protein, maximum 5% crude fibre, 5% crude fat and 7% ash) during the first week of rearing. After that, the chickens were weighed individually (average body weight of 180 ± 0.97 g) and distributed randomly into 4 treatment groups with 7 replicates/pen where each pen contained 14 chickens. The treatment groups included CONT (chicks receiving control feed), UBF (chicks receiving 5% UBF in feed), UBFPRO (chicks receiving 5% UBF in feed plus 0.05% probiotics) and UBFZYM (chicks receiving 5% UBF plus 0.05% multienzyme). Commercial probiotic (Vetafarm, Wagga Wagga, NSW, Australia) containing 1.8 × 10^8^ cfu/g of *Lactobacillus acidophilus*, *Lactobacillus delbrueckii* ssp. *bulgaricus*, *Lactobacillus plantarum*, *Lactobacillus rhamnosus*, *Bifidobacterium bifidum*, *Enterococcus faecium* and *Streptococcus salivarius* ssp. *thermophilus* was used. The commercial multienzyme (Natuzyme, Bioproton Europe Oy, Kaarina, Finland) consisted of cellulose (6000,000 μm/kg), xylanase (10,000,000 μm/kg), β‐glucanase (700,000 μm/kg), protease (3000,000 μm/kg), α‐amylase (700,000 μm/kg), pectinase (70,000 μm/kg), phytase (1300,000 μm/kg), and lipase (5000 μm/kg) was also used in this study.

The chicks were raised in a rice husk–bedded open‐sided broiler house. Throughout the trial, a constant lighting schedule was applied. The feeds (mash form) were prepared to be isocaloric and isonitrogenous, and they met the Indonesian National Standard for broiler starter (days 8–21; Table 2) and finisher feed (days 22–38; Table 3). The chicks were given Newcastle disease (ND) and infectious bronchitis vaccines by spray soon after hatching. The ND vaccination was also given to the chicks at the age of 18. Body weight (individually weighed), feed intake and feed conversion ratio (FCR) of broilers were recorded weekly. Feed intake was measured as the difference between the amount of feed offered and the remaining feed, whereas the FCR was calculated by dividing feed intake by the weight gain of broilers. On day 38, one male chick from each pen (seven chicks per treatment group) were slaughtered, defeathered and eviscerated. The weight (empty) of each internal organ was determined. The carcass weight and commercial cuts of broiler were determined and meat samples (from breast and thigh) were collected for the measurement of meat colour and pH of meats. The colour of the meat was checked using a digital colour meter running on Mac OS X (set to CIE Lab). The colour was represented by the L* (lightness), a* (redness) and b* (yellowness) values. The pH values of meats were determined using pH meter (Portable pH Meter OHAUS ST300).

**TABLE 2:** Ingredients and nutritional contents of starter feed (days 8–21)

**Items (%, unless otherwise noted)**	**CONT**	**UBF**	**UBFPRO**	**UBFZYM**
Yellow maize	56.7	51.1	51.1	51.1
Palm oil	1.15	1.25	1.25	1.25
Soybean meal	39.6	40.1	40.1	40.1
DL‐methionine, 990 g	0.20	0.20	0.20	0.20
Bentonite	0.50	0.50	0.50	0.50
Limestone	0.50	0.50	0.50	0.50
Monocalcium phosphate	0.55	0.55	0.55	0.55
Premix^a^	0.35	0.35	0.35	0.35
Chlorine chloride	0.10	0.10	0.10	0.10
Salt	0.35	0.35	0.35	0.35
Unripe banana flour	–	5.00	5.00	5.00
Calculated chemical constituents				
ME (kcal/kg)^b^	2901	2900	2900	2900
Crude protein (%)	22.0	22.0	22.0	22.0
Crude fibre (%)	5.65	5.61	5.61	5.61
Ca (%)	0.77	0.76	0.76	0.76
P (available) (%)	0.45	0.42	0.42	0.42

Abbreviation: UBF, unripe banana flour.

^a^ Per kg of feed contained 1100 mg Zn, 1000 mg Mn, 75 mg Cu, 850 mg Fe, 4 mg Se, 19 mg I, 6 mg Co, 1225 mg K, 1225 mg Mg, 1250,000 IU vit A, 250,000 IU vit D_3_, 1350 g pantothenic acid, 1875 g vit E, 250 g vit K_3_, 250 g vit B_1_, 750 g vit B_2_, 500 g vit B_6_, 2500 mg vit B_12_, 5000 g niacin, 125 g folic acid and 2500 mg biotin.

^b^ ME (metabolizable energy) was calculated based on formula (Bolton, 1967): 40.81 {0.87 [crude protein + 2.25 crude fat + nitrogen‐free extract] + 2.5}.

**TABLE 3:** Ingredients and nutritional contents of finisher feed (days 22–38)

Items (%, unless otherwise noted)	CONT	UBF	UBFPRO	UBFZYM
Yellow maize	65.0	59.3	59.3	59.3
Palm oil	1.25	1.35	1.35	1.35
Soybean meal	31.2	31.8	31.8	31.8
DL‐methionine, 990 g	0.20	0.20	0.20	0.20
Bentonite	0.50	0.50	0.50	0.50
Limestone	0.50	0.50	0.50	0.50
Monocalcium phosphate	0.55	0.55	0.55	0.55
Premix^a^	0.35	0.35	0.35	0.35
Chlorine chloride	0.10	0.10	0.10	0.10
Salt	0.35	0.35	0.35	0.35
Unripe banana flour	–	5.00	5.00	5.00
Calculated chemical constituents				
ME (kcal/kg)^b^	3001	3000	3000	3000
Crude protein (%)	19.0	19.0	19.0	19.0
Crude fibre (%)	5.75	5.71	5.71	5.71
Ca (%)	0.75	0.74	0.74	0.74
P (available) (%)	0.46	0.44	0.44	0.44

Abbreviation: UBF, unripe banana flour.

^a^ Per kg of feed contained 1100 mg Zn, 1000 mg Mn, 75 mg Cu, 850 mg Fe, 4 mg Se, 19 mg I, 6 mg Co, 1225 mg K, 1225 mg Mg, 1250,000 IU vit A, 250,000 IU vit D_3_, 1350 g pantothenic acid, 1875 g vit E, 250 g vit K_3_, 250 g vit B_1_, 750 g vit B_2_, 500 g vit B_6_, 2500 mg vit B_12_, 5000 g niacin, 125 g folic acid and 2500 mg biotin.

^b^ ME (metabolizable energy) was calculated based on formula (Bolton, 1967): 40.81 {0.87 [crude protein + 2.25 crude fat + nitrogen‐free extract] + 2.5}.

Data were statistically analysed according to a completely randomized design using analysis of variance (SPSS version 16.0). Duncan's multiple range test showed a significant effect of dietary treatment (*p* < 0.05). Orthogonal contrast was also conducted to assess the mean comparison among treatment groups (SPSS version 16.0).

## 3 RESULTS

### 3.1 Growth performance of broiler chickens

Table 4 shows the data on growth performance of broiler chickens during the period of rearing. FCR was lower (*p* < 0.05) in UBF, UBFPRO and UBFZYM than that in CONT chicks during the period of 8–38 and 22–38 days, with no significant difference in body weight, body weight gain and feed intake. There was no substantial effect (*p* > 0.05) of dietary treatments on body weight, body weight gain, feed intake and FCR of broilers during the period of 8–21 days. Orthogonal contrast tests conducted on days 8–38 (Table 5) showed that body weight and body weight gain were different between CONT vs. UBFZYM (*p* < 0.05) and UBF vs. UBFZYM (*p* = 0.05). Moreover, FCR was different (*p* < 0.05) between CONT vs. UBF and UBFPRO vs. UBFZYM.

**TABLE 4:** Growth performance of broiler chickens

Items	CONT	UBF	UBFPRO	UBFZYM	SEM	*p*‐Value
Days 8–21						
BW (g/bird)	652	676	675	691	6.49	0.20
BWG (g/bird)	473	497	495	512	6.49	0.20
FI (g/bird)	694	708	697	752	10.2	0.15
FCR	1.47	1.43	1.41	1.43	0.01	0.41
Days 22–38						
BW (g/bird)	1489	1558	1610	1663	27.0	0.12
BWG (g/bird)	836	881	935	972	23.2	0.18
FI (g/bird)	2621	2370	2358	2404	64.1	0.45
FCR	3.18^a^	2.71^b^	2.53^b^	2.47^b^	0.08	<0.01
Days 8–38						
BW (g/bird)	1489	1558	1610	1663	27.0	0.12
BWG (g/bird)	1309	1378	1430	1484	27.0	0.12
FI (g/bird)	3314	3079	3055	3157	68.8	0.56
FCR	2.53^a^	2.24^b^	2.14^b^	2.13^b^	0.05	<0.01

*Note*: ^a,b^Means in the same row with different superscripts differ significantly (*p* < 0.05).

Abbreviations: BW, body weight; BWG, body weight gain; CONT, chicks receiving control feed; FCR, feed conversion ratio; FI, feed intake; SEM, standard error of the means; UBF, chicks receiving 5% unripe banana flour in feed; UBFPRO, chicks receiving 5% unripe banana flour in feed plus 0.05% probiotics; UBFZYM, chicks receiving 5% unripe banana flour plus 0.05% multienzyme.

**TABLE 5:** Contrasts among dietary treatments on broiler performance at days 8–38

	*p*‐Values		
Contrasts	BW	BWG	FCR
CONT vs. UBF	0.35	0.35	<0.01
CONT vs. UBFPRO	0.10	0.11	<0.01
CONT vs. UBFZYM	0.02	0.02	<0.01
UBF vs. UBFPRO	0.31	0.32	0.86
UBF vs. UBFZYM	0.05	0.05	0.40
UBFPRO vs. UBFZYM	0.29	0.28	0.38

Abbreviations: BW, body weight; BWG, body weight gain; CONT, chicks receiving control feed; FCR, feed conversion ratio; FI, feed intake; UBF, chicks receiving 5% unripe banana flour in feed; UBFPRO, chicks receiving 5% unripe banana flour in feed plus 0.05% probiotics; UBFZYM, chicks receiving 5% unripe banana flour plus 0.05% multienzyme.

### 3.2 Internal organ weights of broiler chickens

There was no substantial difference (*p* < 0.05) among the groups of chicks with regard to the relative weight of internal organs of broilers (Table 6). Yet, there was a tendency that gizzard was higher (*p* = 0.08) in CONT than in UBF chicks. Also, pancreas tended (*p* = 0.09) to be lower in UBFZYM than in CONT birds. Contrast analysis (Table 7) showed that gizzard relative weight was different among CONT vs. UBF (*p* < 0.05), CONT vs. UBFPRO (*p* = 0.09) and UBF vs. UBFZYM (*p* < 0.05). The pancreas relative weight was different between CONT vs. UBF (*p* = 0.09), CONT vs. UBFZYM (*p* < 0.05) and UBFPRO vs. UBFZYM (*p* = 0.09). Contrast analysis also showed no significant difference among treatment groups with regard to the relative weight of heart, liver, proventriculus, duodenum, jejunum, ileum, caeca, abdominal fat, spleen, thymus and *bursa of Fabricius* (data not shown).

**TABLE 6:** Relative weight of internal organs of broiler chickens

Items (% live BW)	CONT	UBF	UBFPRO	UBFZYM	SEM	*p*‐Value
Heart	0.43	0.46	0.46	0.42	0.01	0.66
Liver	2.37	2.28	2.32	2.19	0.06	0.82
Proventriculus	0.52	0.51	0.49	0.51	0.01	0.87
Gizzard	2.14	1.77	1.88	2.02	0.05	0.08
Pancreas	0.29	0.25	0.26	0.23	0.01	0.09
Duodenum	0.98	1.06	0.77	0.84	0.07	0.46
Jejunum	1.09	1.07	1.06	0.86	0.06	0.52
Ileum	0.95	0.94	0.80	0.77	0.06	0.64
Caeca	0.37	0.39	0.49	0.47	0.03	0.31
Abdominal fat	1.11	0.76	0.82	0.88	0.08	0.50
Spleen	0.12	0.12	0.11	0.12	0.01	0.99
Thymus	0.17	0.11	0.19	0.16	0.02	0.20
*Bursa of Fabricius*	0.08	0.07	0.10	0.09	0.01	0.35

Abbreviations: BW, body weight; CONT, chicks receiving control feed; SEM, standard error of the means; UBF, chicks receiving 5% unripe banana flour in feed; UBFPRO, chicks receiving 5% unripe banana flour in feed plus 0.05% probiotics; UBFZYM, chicks receiving 5% unripe banana flour plus 0.05% multienzyme.

**TABLE 7:** Contrasts among dietary treatments on selected internal organ weights

	*p*‐Values	
Contrasts	Gizzard	Pancreas
CONT vs. UBF	0.02	0.09
CONT vs. UBFPRO	0.09	0.25
CONT vs. UBFZYM	0.42	0.02
UBF vs. UBFPRO	0.27	0.52
UBF vs. UBFZYM	0.02	0.37
UBFPRO vs. UBFZYM	0.21	0.09

Abbreviations: CONT, chicks receiving control feed; UBF, chicks receiving 5% unripe banana flour in feed; UBFPRO, chicks receiving 5% unripe banana flour in feed plus 0.05% probiotics; UBFZYM, chicks receiving 5% unripe banana flour plus 0.05% multienzyme.

### 3.3 Carcass traits, pH values and colour of broiler meats

The data on carcass and commercial cuts of broilers are presented in Table 8. There was no difference (*p* > 0.05) between carcass and commercial proportion of broilers. However, contrast analysis (Table 9) showed the difference (*p* < 0.05) in eviscerated carcass between UBF and UBFPRO groups.

**TABLE 8:** Carcass and commercial cuts of broiler chickens

Items	CONT	UBF	UBFPRO	UBFZYM	SEM	*p*‐Value
Eviscerated carcass (% live BW)	68.6	70.3	66.3	69.8	0.62	0.10
	% Eviscerated carcass					
Breast	34.9	36.0	34.2	34.4	0.70	0.83
Wings	12.0	11.5	12.4	11.0	0.25	0.23
Thigh	15.8	15.8	14.9	15.8	0.27	0.58
Drumstick	14.4	14.6	14.8	14.0	0.30	0.87
Back	22.9	22.2	23.8	24.8	0.60	0.49

Abbreviations: BW, body weight; CONT, chicks receiving control feed; SEM, standard error of the means; UBF, chicks receiving 5% unripe banana flour in feed; UBFPRO, chicks receiving 5% unripe banana flour in feed plus 0.05% probiotics; UBFZYM, chicks receiving 5% unripe banana flour plus 0.05% multienzyme.

**TABLE 9:** Contrasts among dietary treatments on eviscerated carcass

Contrasts	*p*‐Values
CONT vs. UBF	0.31
CONT vs. UBFPRO	0.18
CONT vs. UBFZYM	0.48
UBF vs. UBFPRO	0.04
UBF vs. UBFZYM	0.77
UBFPRO vs. UBFZYM	0.14

Abbreviations: CONT, chicks receiving control feed; UBF, chicks receiving 5% unripe banana flour in feed; UBFPRO, chicks receiving 5% unripe banana flour in feed plus 0.05% probiotics; UBFZYM, chicks receiving 5% unripe banana flour plus 0.05% multienzyme.

There was a notable effect (*p* < 0.05) of dietary treatments on b* values of thigh meats, in which UBFPRO had lower b* values than that of CONT but did not differ from that of UBF and UBFZYM (Table 10). Contrast analysis (Table 11) further showed that b* values of thigh meats differed (*p* < 0.05) between CONT and UBFPRO groups. pH values of breast and thigh meats did not differ (*p* < 0.05) across treatment groups. The L*, a* and b* values of breast meats did not vary among the groups. Similarly, L* and a* values of thigh meats were not affected (*p* > 0.05) by the dietary treatments.

**TABLE 10:** pH values and colour of breast and thigh meats of broiler chickens

Items	CONT	UBF	UBFPRO	UBFZYM	SEM	*p*‐Value
Breast						
pH	5.97	5.96	5.98	5.97	0.01	0.34
L*	56.9	57.9	56.2	58.0	0.54	0.60
a*	6.15	4.69	7.03	6.08	0.57	0.60
b*	13.4	11.7	11.6	10.5	0.53	0.30
Thigh						
pH	6.01	6.01	6.04	6.01	0.01	0.36
L*	57.0	56.0	55.0	58.3	0.65	0.32
a*	7.27	6.74	6.04	5.34	0.53	0.63
b*	12.4^a^	11.4^ab^	8.60^b^	9.59^ab^	0.54	0.04

*Note*: ^a,b^Means in the same row with different superscripts differ significantly (*p* < 0.05). L*, lightness values; a*, redness values; b*, yellowness values.

Abbreviations: CONT, chicks receiving control feed; UBF, chicks receiving 5% unripe banana flour in feed; SEM, standard error of the means; UBFPRO, chicks receiving 5% unripe banana flour in feed plus 0.05% probiotics; UBFZYM, chicks receiving 5% unripe banana flour plus 0.05% multienzyme.

**TABLE 11:** Contrasts among dietary treatments on yellowness values of thigh broiler meats

Contrasts	*p*‐Values
CONT vs. UBF	0.47
CONT vs. UBFPRO	0.01
CONT vs. UBFZYM	0.06
UBF vs. UBFPRO	0.06
UBF vs. UBFZYM	0.22
UBFPRO vs. UBFZYM	0.29

Abbreviations: CONT, chicks receiving control feed; UBF, chicks receiving 5% unripe banana flour in feed; UBFPRO, chicks receiving 5% unripe banana flour in feed plus 0.05% probiotics; UBFZYM, chicks receiving 5% unripe banana flour plus 0.05% multienzyme.

## 4 DISCUSSION

The use of the underconsumed banana cultivars as functional feed ingredients was subjected not only to increase the economic value of the banana cultivar, but also to improve the health and growth performance of broilers. It was clear in the current study that the use of unripe banana (“Norowito” cultivar) flour improved the FCR of broilers throughout the study period. The improvement in FCR of broiler with feeding UBF may be attributed to the functional properties of UBF, including prebiotic activity (Munita et al., 2022). Indeed, literature revealed that prebiotics can improve the microbial balance and thus digestive and absorptive functions of broiler intestine (Sugiharto, 2016). The antioxidative property of UBF seemed also to be responsible for the improvement in the physiological conditions, which eventually enhanced the energy allocation for growth. At days 22–38, the FCR of the entire groups of broilers was substantially higher than the normal FCR of commercial broilers at the same period. The high temperature (31.0 ± 1.91°C) and humidity (71.0% ± 7.81%) inside the broiler house during the rearing (days 22–38) were most likely to induce heat stress in the chickens. To compensate for the heat stress, the chicks tended to allocate more energy for the maintenance rather than for growth, leading to higher FCR. Interesting finding was observed during days 22–38, in which FCR was improved with feeding UBF. It was inferred that antioxidative property of UBF may be beneficial in alleviating the detrimental effect of heat stress by scavenging the excessive free radicals during the heat stress condition that in turn improve the physiological condition of broilers. The latter condition may confirm the efficacy of UBF in improving the FCR of broilers, especially during the finisher period.

The use of probiotic or multienzyme in combination with UBF was expected to exert synergistic effect between these active ingredients and thus further improve the broiler performance. Contrast analysis showed that the use of multienzyme together with UBF resulted in more superior effect on body weight and body weight gain of broilers as compared to the effect of UBF alone. Study suggested that enzyme supplementation was associated with the improved intestinal microbial ecosystem, morphology as well as digestibility of broilers (Van Hoeck et al., 2021). These functional effects of enzymes may therefore support the prebiotic effect of UBF in improving the nutrient utilization of broilers. In line with this present study, Shang and Kim (2017) also confirmed that prebiotics can work synergistically with enzymes in improving broiler production performance. In contrast to enzymes, the application of probiotics in combination with UBF did not exert any additional effect on the growth of broilers when compared with the application of UBF alone. So far, the definite reason for such a condition remains unclear. However, the inconsistent effect of probiotics (Soumeh et al., 2021; Sugiharto, 2016) seemed to explain the lack of synergistic effect of probiotics and UBF on broiler performances in this current study.

Data in our present study showed a clear tendency that feeding UBF reduced the relative weight of gizzard of broilers. Previous study suggested that the high content of insoluble fibre in diet may implicate further grinding activity leading to further muscular layer development and hence increasing gizzard size (Rungcharoen et al., 2013). Taken the latter study into consideration, the high content of soluble fibre (Falcomer et al., 2019) and carbohydrates (Munita et al., 2022) in UBF may therefore reduce grinding activity and thus prevent the further muscular layer development of gizzard. Different from the UBF and UBF plus probiotics, the use of multienzyme in combination with UBF did not exert lowering effect on gizzard weight. Indeed, contrast analysis showed that UBFZYM chicks had higher gizzard weight as compared to UBF chicks. In line with our study, Amerah et al. (2015) reported that enzyme (endoxylanase and β‐glucanase) supplementation increased the relative weight of gizzard of broilers in their study. In general, enzyme has been reported to improve the digestibility of nutrients, including fibre, and thereby reduce the grinding activity and gizzard weight (Rungcharoen et al., 2013). In this regard, the attribution of multienzyme to increasing the relative weight of gizzard seemed to be inappropriate. Yet, one study by Bedford and Schulze (1998) revealed that xylanase indirectly stimulates the function of the gizzard and proventriculus (Bedford & Schulze, 1998). Owing to this, it is tempting to speculate that exogenous enzyme supplementation may enhance the growth and development (muscular layer development) of gizzard irrespective of the grinding activity of the gizzard as discussed previously.

Our current study revealed that the relative weight of pancreas tended to decrease with feeding UBF. Previous study by Abdel‐Raheem and Abd‐Allah (2011) showed that feeding prebiotic mannan oligosaccharide reduced the weight of pancreas. Similarly, Fallah and Rezaei (2013) noticed a reduced pancreas weight when feeding commercial prebiotics to broiler chickens. Considering that UBF is rich in prebiotic, the reduced pancreas weight with feeding UBF in this study could therefore be understood. The rationale and mechanism by which prebiotics affected the weight of pancreas remain unclear. Yet, Iji et al. (2001) revealed that prebiotics may improve the structures and activities of pancreatic enzymes, as a consequence, less quantities of enzyme are needed for the chemical digestion of feeds. The latter condition seemed to be attributed to the reduced pancreatic tissue activity (in producing pancreatic enzymes), thus lowering pancreas organ weight. It is interesting to see in this study that the use of multienzyme in combination with UBF resulted in the most substantial reduction in pancreas weight as compared to that of control. In accordance with our data, the relative weight of pancreas decreased when the exogenous glucanase (Moran, 1985) or protease (Nastain et al., 2021) was supplemented to the diets of broilers. It was most likely that exogenous enzymes improved chemical digestion process in the gastrointestinal tract, while reducing the activity of the pancreas in producing the respective enzymes (Moran, 1985; Nastain et al., 2021). The less activity of pancreas may thus be attributed to the reduced pancreatic tissue weight.

Data in our present study showed no substantial effect of dietary treatments on the carcass traits of broiler chickens. However, contrast analysis showed that UBFPRO had lower eviscerated carcass than UBF group, suggesting the lowering effect of probiotics on the carcass weight of broilers. Our finding was in contrast to most of the studies revealing the enhancing effect (Parsa et al., 2018) or no effect (Tang et al., 2021) of probiotics on eviscerated carcass of broilers. However, our finding was in line with Rehman et al. (2020) showing the reduced dressing percentage of broilers with the administration of commercial probiotic (Protexin). The rationale for the lowering effect of probiotics on carcass percentage remains unknown so far. We speculated that probiotics may lower the fat content of broilers (abdominal fat and subcutaneous fat beneath the skin) as reported by Park et al. (2016) when feeding *Lactobacillus sakei* Probio‐65. Such lower fat content may therefore reduce the carcass percentage of broilers. However, our inference should be taken with caution as there was no substantial difference in the abdominal fat content between UBF and UBFPRO chicks. Moreover, we did not measure the subcutaneous fat beneath the skin of broilers in this present study.

The high lightness and yellowness as well as low redness values have been attributed to the pale‐soft‐exudative (PSE) condition in broiler meats (Adzitey & Nurul, 2011). Particularly with respect to the yellowness values, the thigh meats of UBFPRO and UBFZYM had lower yellowness values as compared to that of CONT. Considering the absent effect of UBF on the yellowness values of thigh meats, our data therefore suggested the preventing effect of probiotics or exogenous enzymes from the PSE condition on broiler meats. In agreement with our inference, Abdurrahman et al. (2016) reported that probiotic (*Lactobacillus* sp.) treatment decreased the yellowness values of broiler meats. So far, the mechanism by which the probiotics may reduce the yellowness of broilers meats remains unclear, but Abdurrahman et al. (2016) confirmed that probiotics may lower the fat content in meats, resulting in less yellow of broiler meats. In accordance with probiotics, Szymczyk et al. (2007) showed the reduced fat content in broiler meats with exogenous enzyme administration. The latter condition may therefore be responsible for the lower yellowness values of meat in UBFZYM group. With regard to the prebiotic effect of UBF on the yellowness values of thigh meat, Tavaniello et al. (2018) did not see any substantial effect of prebiotics on the lightness and yellowness indexes of broiler meats in their study.

## 5 CONCLUSIONS

Dietary inclusion of 5% UBF with or without probiotic and multienzyme improve FCR, without negatively affecting the carcass characteristics of broilers.

## AUTHOR CONTRIBUTIONS

Sugiharto Sugiharto and Tugay Ayasan contributed to the design of the study and manuscript preparation. Oktafia Munita performed the experiments and contributed significantly to the analysis and manuscript preparation. Turrini Yudiarti, Endang Widiastuti, Hanny Indrat Wahyuni, Tri Agus Sartono and Ikania Agusetyaningsih performed the experiments.

## CONFLICT OF INTEREST

We did not have any conflict of interest.

### ETHICS STATEMENT

The Animal Ethics Committee of the Faculty of Animal and Agricultural Sciences, Universitas Diponegoro, approved the in vivo experiment (No. 58‐04a/A‐5/KEP‐FPP).

### PEER REVIEW

The peer review history for this article is available at https://publons.com/publon/10.1002/vms3.1070.

## Data Availability

The data obtained during the study are available from the corresponding author on request.
